# Reduced MMN-indexed auditory change detection in a non-clinical high schizotypy sample

**DOI:** 10.1017/pen.2024.3

**Published:** 2024-09-26

**Authors:** Jenna N. Bissonnette, T-Jay Anderson, Lauren Ross, Ashley M. Francis, Kaitlyn Napier, N. Will Shead, Derek J. Fisher

**Affiliations:** 1 Department of Psychology & Neuroscience, Dalhousie University, Halifax, Nova Scotia, Canada; 2 Department of Psychology, Mount Saint Vincent University, Halifax, Nova Scotia, Canada; 3 Department of Psychiatry, Dalhousie University, Halifax, Nova Scotia, Canada

**Keywords:** electroencephalography, event-related potential, impulsivity, mismatch negativity, schizotypy, sensation seeking

## Abstract

Schizotypal traits include abnormalities in cognition, behavior, and interpersonal relationships that are similar, yet less severe than psychotic symptomology. It is estimated that approximately 5% of the general population displays psychotic symptoms and experiences that can be considered schizotypal in nature, but there is little research examining the neurological correlates of these traits. The mismatch negativity (MMN) event-related potential is an objective measure of auditory change detection derived from electroencephalography. The current study contributes to the limited body of evidence examining the neurobiological underpinnings of schizotypy in a non-clinical sample using the MMN. Participants were recruited from the general population and divided into high and low-schizotypy groups for comparison. Individuals with high schizotypal traits displayed reduced MMN amplitudes in response to frequency and location deviants, and longer MMN latencies in response to location deviants. Specific sub-traits of schizotypy were uniquely related to frequency and location amplitudes, suggesting the previously reported inconsistencies in the literature may be due to diverse samples and differing deviant tone types. Finally, impulsivity and sensation-seeking likely contributed to the slower processing seen in location deviance detection. Ultimately, the current results provide evidence that the neurobiological abnormalities seen in clinical populations of schizotypal personality disorder and psychosis also extend to non-clinical populations.

## Understanding schizotypy and dimensional approaches to defining schizophrenia

Schizophrenia is a psychotic disorder with a global prevalence of 1% that is characterized by positive (e.g., hallucinations, delusions), negative (e.g., anhedonia, avolition), and disorganized (DO; e.g., odd speech/behavior) symptoms (American Psychiatric Association, 2022). The current diagnostic classification system for psychotic disorders like schizophrenia is categorical in that it requires the presence of several symptoms to surpass a threshold to receive a diagnosis. However, dimensional approaches to conceptualizing psychotic illness have been growing in popularity (Allardyce et al., 2007; Nelson et al., 2013; Peralta & Cuesta, 2007). Within dimensional conceptualizations, psychosis-like symptoms that do not surpass a diagnostic threshold can either be representative of prodromal phases of psychosis that indicate risk, or they may simply represent a spectrum of personality traits within the general population (Raine, 2006). Within the general population, these sub-clinical psychosis-like symptoms and experiences can be referred to as schizotypal traits. It is estimated that approximately 5% of the general population display these sub-clinical psychosis symptoms and/or psychotic experiences (Van Os et al., 2009).

Individuals with high levels of schizotypal traits are at greater risk of being diagnosed with schizotypal personality disorder (SPD; American Psychiatric Association, 2013). The symptoms of SPD can be divided into three categories: cognitive perceptual (e.g., paranoid ideation, magical thinking, unusual perceptual experiences, ideas of reference (IR)), interpersonal (e.g., few close friends, social anxiety (SA), constricted affect), and DO (e.g., odd speech/behaviors; Raine, 2006). These three categories directly relate to the symptom categories within schizophrenia, with the main difference being the absence of sensory hallucinations in SPD. Accordingly, individuals with schizophrenia typically score higher on measures of schizotypy (Camisa et al., 2005), and those with high levels of schizotypal traits are more likely to develop a psychotic illness than those without (Kwapil et al., 2013). Further, individuals with high levels of self-reported schizotypy show some similar but less pronounced neurobiological abnormalities compared to those with schizophrenia (Mohanty et al., 2005).

Importantly, there is a distinct difference between the presence of schizotypal traits and psychosis; schizotypal traits alone are not necessarily indicative of dysfunction. Therefore, there lies an inherent value within schizotypy research to determine what differentiates, on a neurobiological level, someone with healthy psychological function and schizotypal traits from someone with psychotic illness (Nelson et al., 2013). Achieving a better understanding of the neurological similarities and differences between individuals with psychosis and individuals with high levels of schizotypal traits in the general population will provide more insight into the validity of adopting a dimensional approach to defining psychosis, and will also provide insight into the effects of psychotic-like features on the cortex in the absence of antipsychotic medication.

## The mismatch negativity in schizophrenia and schizotypy

Using electroencephalography (EEG), we can objectively measure brain responses that may underlie psychosis and schizotypy. The mismatch negativity (MMN) is one brain response that occurs in the frontotemporal region approximately 100–250 milliseconds following a detectable change in the auditory environment and can be conceptualized as a general marker of auditory cortex functioning (Näätänen, 2003). MMN generation has high reliability (Recasens & Uhlhaas, 2017; Tervaniemi et al., 1999; Wang et al., 2021) and is dependent on glutamate binding to N-methyl-D-aspartate receptors in the cortex (Umbricht et al., 2000). Given the robustly reported alterations to the glutamatergic system in psychosis (Bissonnette et al., 2022; Marsman et al., 2013; Merritt et al., 2016), an altered MMN has been hypothesized to be a potential marker of psychosis risk. Furthermore, glutamate alterations have also been reported in some high schizotypy groups (Ford et al., 2017; Kozhuharova et al., 2021), but not others (Chen et al., 2022; Modinos et al., 2010), suggesting people with high levels of schizotypal traits may display similar but less pronounced (i.e., not as robustly reported) neurochemical abnormalities that underlie the MMN, and therefore would display similar but less pronounced abnormalities in the MMN response.

Research thus far has identified reduced MMN amplitudes in individuals with chronic (Bodatsch et al., 2011; Erickson et al., 2016; Fisher et al., 2012; Michie, 2001; Umbricht et al., 2003) and early phase (Fisher et al., 2019; Riel et al., 2022; Rudolph et al., 2015) psychosis. In SPD, reports of the MMN response have been mixed, with one report of higher MMN amplitudes in response to frequency deviants (Liu et al., 2007) and one report of reduced amplitudes (Niznikiewicz et al., 2009). Few studies have explored the relationship between the MMN and degree of schizotypal traits in non-clinical populations. Of the existing reports, one found those with high schizotypal traits had reduced MMN amplitudes compared to those with low schizotypal traits (Baldeweg et al., 1999). More recently, Donaldson et al. (2021) found greater schizotypal traits were significantly related to reduced MMN amplitudes in response to a duration deviant. Additionally, there has been one report of no relationship between schizotypal traits and duration MMN amplitudes (Broyd et al., 2016). However, given the paucity of research in non-clinical samples, more research is needed to determine how the MMN response relates to schizotypy in the general population.

## The current study

To add to the limited body of evidence, the current study employed a multi-feature MMN paradigm to explore the MMN in a non-clinical sample of individuals with high schizotypal traits. This paradigm presents five distinct deviant tone types to elicit an MMN and therefore allows for a more comprehensive picture of auditory change detection abilities (Näätänen et al., 2004). Due to the associations between schizotypal traits, impulsivity, and sensation seeking (Del Giudice et al., 2014), we also examined how impulsivity and sensation seeking related to the MMN response in our sample.

## Methods

### Participants

All participants (*N* = 31) were between the ages of 18–42, were fluent in English, right-handed, and had normal or corrected-to-normal vision and hearing. Participants were recruited within Nova Scotia through online advertisements and word-of-mouth. Participants were excluded if they met any of the following criteria: history of head injury leading to concussion or loss of consciousness within the past six months, diagnosis of a neurodevelopmental disorder or a neurological condition (e.g., epilepsy), current or regular medication use (apart from hormonal contraceptives), received electroconvulsive therapy within the past year, or a self-reported diagnosis of a severe mental illness (i.e., bipolar disorder or schizophrenia as per the the Diagnostic and Statistical Manual of Mental Disorders, Fifth Edition). Because previous research has shown a high level of concurrent mental illness diagnoses within individuals with high schizotypal traits (Lewandowski et al., 2006), this study did not exclude participants if they had a self-reported diagnosis of depression or anxiety to ensure the generalizability of the study to the population of interest.

### Procedure

Upon arrival, participants completed informed consent procedures followed by a series of questionnaires and the EEG recording. All sessions took place between 11 AM and 1 PM to control for time of day effects (Hines, 2004). Participants were asked to abstain from drugs and medication (apart from oral contraceptives) beginning midnight the night before their testing session. Verbal confirmation of abstinence was obtained upon arrival at the laboratory. Study procedures were conducted following clearance from the Mount Saint Vincent University Research Ethics Board (REB #2018-010).

### Questionnaires

#### Schizotypal personality questionnaire – brief revised

The Schizotypal Personality Questionnaire – Brief Revised (SPQ-BR) was used to measure schizotypal traits at the time of testing. The SPQ-BR is a 32-item self-report scale that is validated in a non-clinical university sample (Cohen et al., 2010). Participants rated themselves on a five-point Likert scale, where 0 = “strongly disagree” and 4 = “strongly agree”. Higher scores indicated higher schizotypal traits. This scale can be further divided into four subscales: cognitive perceptual (CP), social anxiety (SA), disorganized (DO), and ideas of reference (IR) based on a previously conducted factor analysis (Cohen et al., 2010).

#### Sensation seeking scale form V

The Sensation Seeking Scale Form V (SSS-V) is a self-report measure comprised of 40 forced-choice items surrounding disinhibition, boredom susceptibility, and thrill and adventure seeking (Zuckerman et al., 1978). The SSS-V has been psychometrically validated in a non-clinical university sample (Roberti et al., 2003). Total SSS-V scores were summed for each participant.

#### Barratt impulsiveness scale

The Barratt Impulsiveness Scale (BIS) is a 30-item self-report measure used to assess impulsive traits and behaviors (Patton et al., 1995) that is validated in non-clinical, inpatient, and forensic samples (Patton et al., 1995). Participants indicate on a 4-point Likert scale (1 = never/rarely to 4 = almost always/always) how frequently they exhibit each behavior. An overall total BIS score was calculated for each participant.

### EEG recording

EEG recordings were completed using an electrode cap with Ag^+^/Ag^+^-Cl- ring electrodes at thirty-two scalp sites according to the 10–20 system of electrode placement, including three midline sites (frontal [F_z_], central [C_z_], parietal [P_z_]), three left hemisphere (frontal [F_3_], central [C_3_], temporal [T_7_]) and three right hemispheres (frontal [F_4_], central [C_4_], temporal [T_8_]). Electrodes placed on the right and left mastoid, as well as on the tip of the nose, served as reference and ground channels respectively. Electro-oculogram activity was recorded from supra-/sub-orbital and external canthi sites via bipolar channels. All electrode impedances were below 10kΩ and all electrical activity was recorded using BrainVision Recorder software with an amplifier bandpass of DC to 100 Hz and digitized at 500 Hz. Data were then stored on a hard drive for offline analysis using the BrainVision Analyzer software.

Offline data processing included applying filters from 0.1 to 20 Hz with a notch filter at 60 Hz. Data was then segmented 100 ms before and 700 ms after each deviant tone. Ocular correction using the Gratton and Coles method (Gratton et al., 1983) was completed using horizontal electrooculogram (HEOG) and vertical electrooculogram (VEOG) channels. A baseline correction was completed using 100 ms before each tone. Artifact rejection was done for any epoch with neuroelectric activity exceeding 75 µV. Averages were taken for each tone and a subtraction waveform for each deviant tone was computed by point-by-point subtraction of the standard. MMN amplitudes for each deviant type were taken as the peak voltage (±8 ms) of the subtraction waveform between 100 and 250 ms at F3, Fz, F4, C3, Cz, and C4. MMN latency was the time of peak amplitude at Fz.

### Multi-feature mismatch negativity paradigm

**Figure 1.**
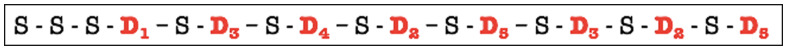
Multi-feature mismatch negativity paradigm. *Note:* The above figure represents the auditory tones presented during the multi-feature MMN paradigm. The black “S” letters represent a presentation of a standard tone, and each red “D” letter represents a presentation of one of the five deviant tone types.

Within the multi-feature MMN paradigm, standard stimuli were tones of 75 ms duration (5 ms rise/fall) that were composed of three sinusoidal partials of 500, 1000, and 1500 Hz. The deviant tones were identical to the standard tones in all physical attributes except where stated. There were five deviant tone types including duration, frequency, intensity, perceived location, and continuity (i.e., a gap in the middle of the tone). The duration deviant was 25 ms (5 ms rise/fall). Half of the frequency deviants were 10% higher (composed of 550, 1100, and 1650 Hz partials) while the other half were 10% lower (composed of 450, 900, and 1350 Hz partials). Half of the intensity deviants were at 60 dB while the other half were at 80 dB. The perceived difference between the standard tone and the location deviant was approximately 90° and was achieved by creating a time difference of 800 μs for half of the location deviants to the left channel and half of the deviants to the right. The gap deviant was created by removing 7ms (including 1 ms rise and fall) from the middle of the standard stimulus. See Figure 1 for a visual representation of the multi-feature paradigm.

Participants watched a silent neutral film (Disney’s Fantasia) while tones played through a set of over-ear headphones. The stimuli were presented binaurally in three 5-minute blocks and participants were given short breaks (1–2 min) between each block. At the beginning of each block, the standard tone was presented 15 times consecutively. This was followed by a sequence in which the standard tone was every second tone (*P* = 0.5) and every other tone was 1 of 5 deviants (*P* = 0.1 each). Each deviant category was presented once every 5 deviants and two of the same deviant type were never presented consecutively. The interstimulus interval was 500 ms. In each 5-minute block there were a total of 600 stimuli presented (1800 stimuli over all 3 blocks) and each deviant type was presented 180 times.

### Statistical analysis

**Figure 2.**
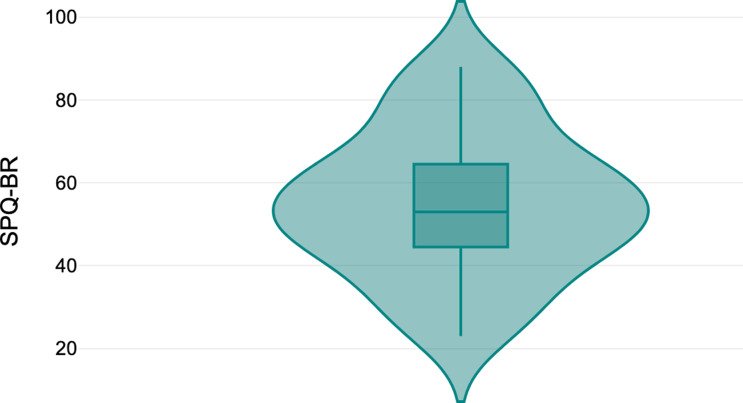
Distribution of Schizotypal Personality Questionnaire – Brief Revised (SPQ-BR) scores in full sample. *Note:* The above violin plot displays the distribution of SPQ-BR scores in the full sample of participants.

All statistical analyses were completed using the Statistical Package for the Social Sciences (SPSS 27; SPSS Inc., Chicago, IL). High and low schizotypy groups were generated based on cut-offs used in previous research (Cohen et al., 2012; Dinzeo & Thayasivam, 2021; Francis et al., 2023) where SPQ-BR scores falling below the mean were designated as low, and SPQ-BR scores falling above the mean were designated as high (see Figure 2 for a visual representation of the distribution of SPQ-BR scores). There were 16 individuals in the high schizotypy group and 15 individuals in the low schizotypy group. As expected, the high schizotypy group (HSTPY) was significantly higher in SPQ-BR total scores (*t*[29] = –7.26, *p* < .001), as well as in the CP (*t*[29] = –6.90, *p* < .001), IR (*t*[29] = –4.78, *p* < .001), DO (*t*[29] = –4.73, *p* < .001), and SA (*t*[29] = –3.53, *p* < .001) subscales. Independent samples t-tests using a Sidak correction were used to explore any potential differences between high and low schizotypy groups in age, sensation seeking, and impulsivity. Further, group differences in sex and comorbid diagnosis status were explored using Chi-Square tests of independence.

**Figure 3.**
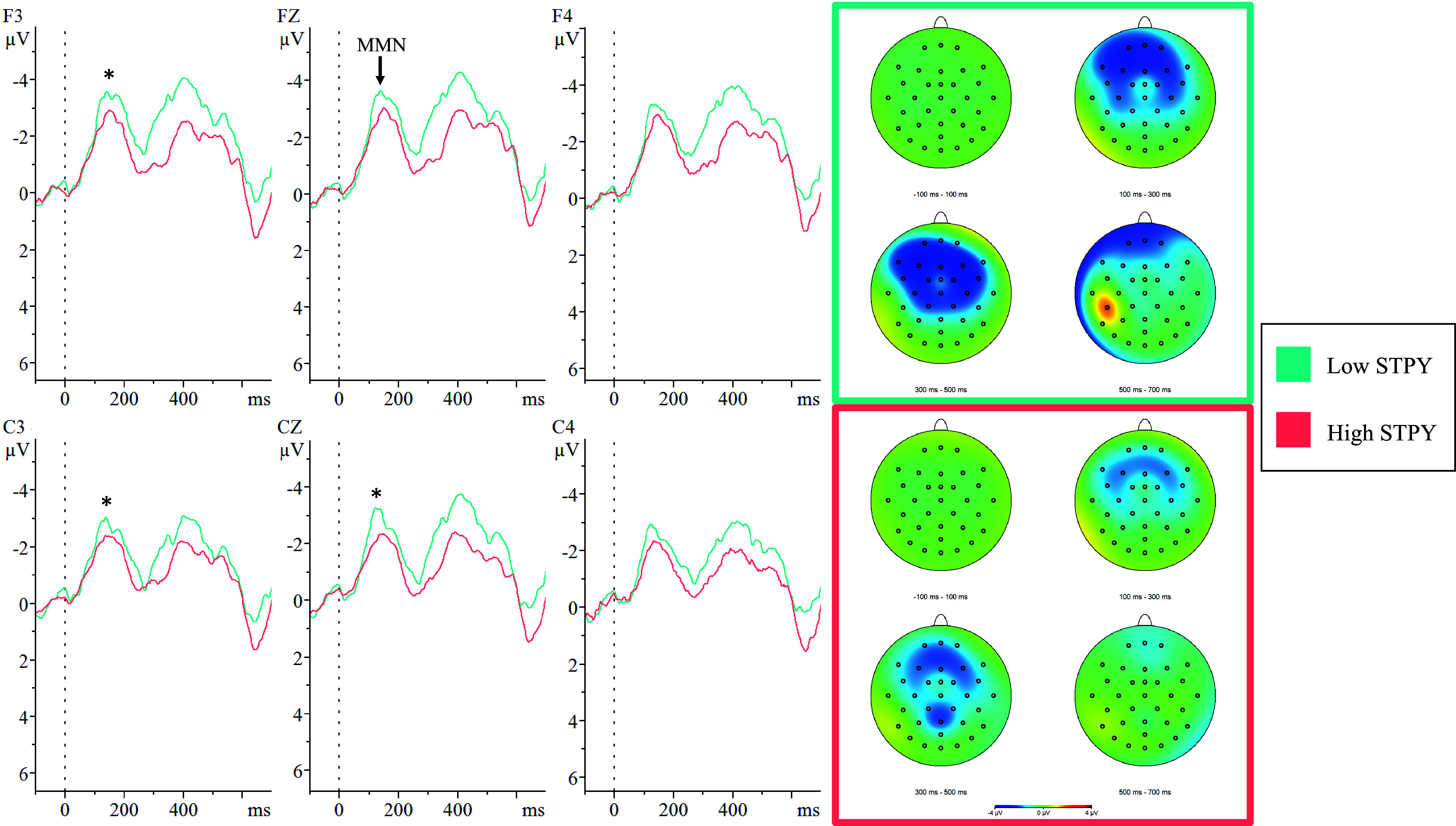
Group comparisons of mean mismatch negativity (MMN) amplitudes in response to frequency deviants. *Note:* The above figure displays the grand averaged MMN waveforms at electrode sites F3, Fz, F4, C3, Cz, and C4 as well as the topographic distributions elicited by a frequency deviant. *Indicates a significant difference between the low schizotypy group (Low STPY; teal) and high schizotypy group (High STPY; coral) at the *p* < .05 level.

**Figure 4.**
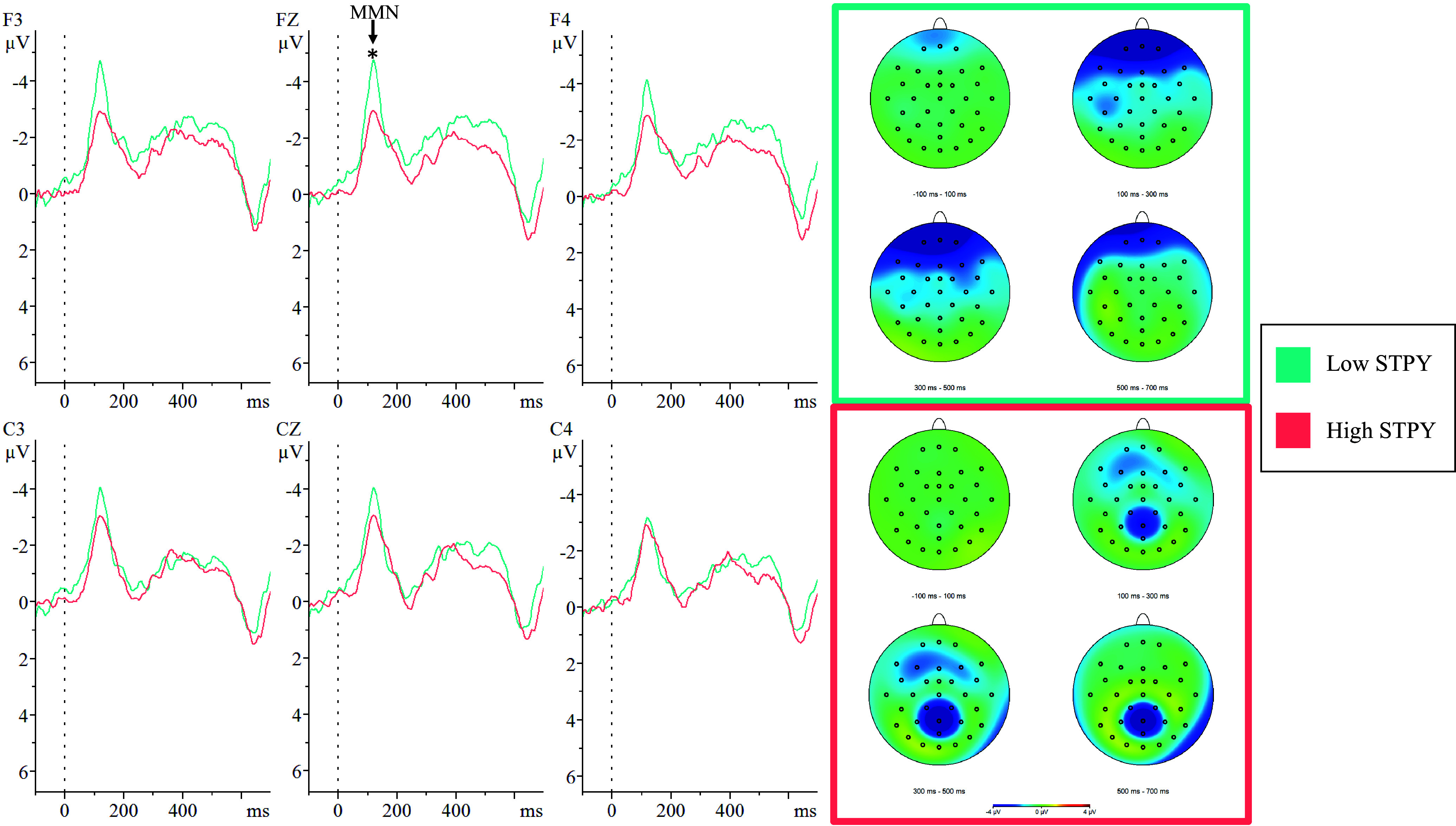
Group comparisons of mean mismatch negativity (MMN) amplitudes in response to location deviants. *Note:* The above figure displays the grand averaged MMN waveforms at electrode sites F3, Fz, F4, C3, Cz, and C4 as well as the topographic distributions elicited by a location deviant. *Indicates a significant difference between the low schizotypy group (Low STPY; teal) and high schizotypy group (High STPY; coral) at the *p* < .05 level.

Separate repeated-measures analyses of variance (ANOVAs) were carried out for MMN amplitudes to each deviant tone type. The ANOVAs included the between-subjects factor of group (2 levels: low schizotypy, high schizotypy) and the within-subjects factors of laterality (3 levels: right, midline, left) and region (2 levels: frontal and central). This allowed us to examine MMN amplitudes in response to the five deviant tone types at electrode sites F3, Fz, F4, C3, Cz, and C4. Greenhouse-Geisser corrected values were used to account for the violation of normality within the EEG data. Separate independent samples t-tests with a Sidak correction were performed to assess group differences between low and high schizotypy groups and MMN latencies at electrode site Fz for each deviant tone type.

Bivariate Spearman’s rho non-parametric correlations between SPQ-BR total scores and subscale scores and MMN amplitudes and latencies at the site of maximal amplitude (Fz) for each deviant tone type were completed in the total sample as well as in both the high and low schizotypy groups separately. Finally, relationships between MMN amplitudes and latencies at Fz for each deviant tone type and measures of impulsivity (BIS) and sensation seeking (SSS-V) were explored with bivariate Spearman’s rho non-parametric correlations in the high and low schizotypy groups separately. The full dataset is available at https://doi.org/10.17605/OSF.IO/M2FDB.

## Results

### Group comparisons

The HSTPY and low schizotypy group (LSTPY) did not differ significantly in age, sex, sensation seeking, or the number of individuals within the group who had a self-reported diagnosis of anxiety or depression. HSTPY (*M* = 63.25, *SD* = 9.43) were significantly higher compared to LSTPY (*M* = 56.47, *SD* = 8.50) in impulsivity measured by the BIS (*t*[29] = –2.10, *p* = .045, *d* = 0.76). See Table 1 for a full report of group comparisons.

**Table 1.** Group comparisons of demographic variables and questionnaire scores

	Low STPY (*n* = 15)	High STPY (*n* = 16)	Test statistic *Chi-square(df)/t(df)*	*p*-value	*Cohen’s d*
Sex (M/F)	7/8	7/9	0.03 (1)	.870	–
Presence of a self-reported anxiety or depression diagnosis (PRESENT/ABSENT)	4/11	6/10	0.42 (1)	.416	–
Age	25.60 (±4.72)	24.94 (±6.42)	0.33 (29)	.747	0.12
SPQ-BR Total Score	40.73 (±4.65)	68.13 (±11.32)	**–7.23 (29)**	**<.001** *	**3.17**
SPQ-BR Cognitive Perceptual	18.53 (± 4.94)	30.56 (±4.76)	**–6.90 (29)**	**<.001** *	**2.48**
SPQ-BR Ideas of Reference	7.53 (±2.64)	12.00 (±2.55)	**–4.78 (29)**	**<.001** *	**1.72**
SPQ-BR Disorganized	10.00 (±3.42)	17.89 (±5.52)	**–4.73 (29)**	**<.001** *	**1.72**
SPQ-BR Social Anxiety	4.67 (±2.38)	7.88 (±2.66)	**–3.53 (29)**	**<.001** *	**1.27**
Sensation Seeking Scale	19.13 (±6.23)	19.24 (±6.52)	–0.05 (29)	.962	0.02
Barratt Impulsiveness Scale	56.47 (±8.50)	63.25 (±9.43)	**–2.10 (29)**	**.045** *	**0.76**

*Note:* The above table displays the group mean values and standard deviations for each demographic variable (sex, age, and presence of self-reported anxiety or depression) and questionnaire score collected. The *p*-values have been derived from independent samples t-tests (for continuous variables) or chi-square tests of independence (for categorical variables).

* Indicates significant differences at the *p* ≤ .05 level.

### MMN amplitudes

There were no significant main or interaction effects for MMN amplitudes elicited by duration, intensity, or gap deviants.

#### Frequency deviant

There was a significant main effect of group (*F*[29] = 5.93, *p* = .021, *d* = 0.21) where LSTPY (*M* = –3.58 μV, *SD* = 1.91) had higher MMN amplitudes compared to HSTPY (*M* = –2.77 μV, *SD* = 4.94). Significant group-by-site interactions revealed LSTPY (*M*_*left*_ = –3.70 μV, *SD*_*left*_ = 0.93; *M*_*midline*_ = –3.78 μV, *SD*_*midline*_ = 1.26) had higher MMN amplitudes compared to HSTPY (*M*_*left*_ = –2.78 μV, *SD*_*left*_ = 1.28; *M*_*midline*_ = –2.84 μV, *SD*_*midline*_ = 1.36) at left hemisphere (*F*[29] = 7.52, *p* = .010, *d* = 0.82) and midline (*F*[29] = 6.52, *p* = .016, *d* = 0.72) electrode sites. Additionally, a group-by-region interaction revealed that LSTPY (*M* = –3.24 μV, *SD* = 0.91) had higher MMN amplitudes compared to HSTPY (*M* = –2.40 μV, *SD* = 1.04) at the central region (*F*[29] = 6.42, *p* = .017, *d* = 0.86), but this interaction did not reach significance at the frontal region (*F*[29] = 3.66, *p* = .066, *d* = 0.67). Finally, group-by-site-by-region interactions revealed that LSTPY had significantly higher MMN amplitudes at electrode sites F3, C3, and Cz (see Figure 3; Table 2).

**Table 2.** Mean mismatch negativity amplitudes for each deviant tone type

Deviant tone type	Electrode site	Low schizotypy *M* (±*SD*)	High schizotypy *M (*±*SD*)	*F(df)*	*p*-value	*Cohen’s d*
Frequency	**F3**	**–4.06 (±1.19)**	**–3.10 (±1.14)**	**5.18 (29)**	**.030** *	**0.82**
	Fz	–4.07 (±1.08)	–3.21 (±1.36)	3.77 (29)	.062	0.70
	F4	–3.59 (±0.92)	–3.07 (±1.28)	1.72 (29)	.200	0.47
	**C3**	**–3.34 (±0.74)**	**–2.46 (±1.08)**	**6.94 (29)**	**.013** *	**0.95**
	**Cz**	**–3.49 (±0.95)**	**–2.46 (±1.12)**	**7.54 (29)**	**.010** *	**0.99**
	C4	–2.90 (±0.95)	–2.29 (±1.04)	2.88 (29)	.101	0.61
Location	F3	–4.11 (±1.87)	–3.11 (±1.09)	3.39 (29)	.076	0.65
	**Fz**	**–4.22 (±2.01)**	**–3.02 (±1.00)**	**4.50 (29)**	**.043** *	**0.76**
	F4	–3.74 (±1.80)	–2.85 (±0.81)	3.22 (29)	.083	0.64
	C3	–3.65 (±1.67)	–2.77 (±1.08)	3.09 (29)	.089	0.63
	Cz	–3.80 (±1.95)	–2.84 (±1.05)	2.97 (29)	.095	0.61
	C4	–3.06 (±1.80)	–2.53 (±0.97)	1.07 (29)	.310	0.37

*Note:* The above table displays the mean mismatch negativity (MMN) amplitudes in microvolts (μV) for each deviant tone type at electrode sites F3, Fz, F4, C3, Cz, and C4.

* Indicates significant differences between groups at the *p* ≤ .05 level.

#### Location deviant

There was a trend approaching significance (*F*[29] = 3.38, *p* = .076, *d* = 0.30) for a main effect of group where LSTPY (*M* = –3.77 μV, *SD* = 4.16) had significantly higher MMN amplitudes compared to HSTPY (*M* = –2.85 μV, *SD* = 1.28). Followed up to account for electrode region, there was a trend towards a group-by-region interaction where LSTPY (*M* = –4.02 μV, *SD* = 1.28) had higher MMN amplitudes compared to HSTPY (*M* = –2.99 μV, *SD* = 1.38) in the frontal region (*F*[29] = 3.96, *p* = .056, *d* = 0.77). Followed up further, there was a significant group-by-site-by-region interaction (*F*[29] = 4.50, *p* = .043, *d* = 0.76) where LSTPY (*M* = –4.22 μV, *SD* = 2.01) had higher MMN amplitudes compared to HSTPY (*M* = –3.02 μV, *SD* = 1.00) at electrode site Fz (see Figure 4; Table 2).

### MMN latencies

LSTPY (*M* = 123.10 ms, *SD* = 14.20) had significantly shorter MMN latencies than HSTPY (*M* = 141.13 ms, *SD* = 30.86) in response to the location deviant (*t*[29] = –2.07, *p* = .048, *d* = 0.76). There were no significant differences in MMN latencies between groups for any other deviant tone type (see Table 3).

**Table 3.** Mean mismatch negativity (MMN) latencies for each deviant tone type at Fz

Deviant tone type	Low schizotypy *M* (±*SD* **)**	High schizotypy *M (*±*SD* **)**	*t(df)*	*p*-value	*Cohen’s d*
Duration	150.8 (±15.8)	150.4 (±17.4)	0.07 (29)	.944	0.02
Frequency	149.0 (±28.1)	151.8 (±18.6)	–0.32 (29)	.749	0.12
Intensity	147.1 (±32.5)	151.4 (±19.4)	–0.45 (29)	.660	0.16
Location	123.1 (±14.2)	141.1 (±30.9)	**–2.07 (29)**	**.048** *	**0.75**
Gap	162.2 (±15.0)	155.5 (±15.6)	1.22 (29)	.233	0.44

*Note:* The above table displays the MMN latencies in milliseconds (ms) for each deviant tone type. Average values were derived from the site of maximal amplitude (Fz).

* Indicates a significant difference between high schizotypy (STPY) and low STPY groups at the *p* < .05 level.

### Correlations

In the full sample (both LSTPY and HSTPY), higher scores on the CP subscale of the SPQ-BR were related to reduced MMN amplitudes following the frequency deviant (*r* = .363, *p* = .045), and higher scores on the SA subscale of the SPQ-BR were related to reduced MMN amplitudes following the location deviant (*r* = .400, *p* = .026). Furthermore, In HSTPY only, higher scores on the IR subscale of the SPQ-BR were related to reduced MMN amplitudes following a frequency deviant (*r* = .530, *p* = .035). Finally, in LSTPY only, higher scores on the DO subscale of the SPQ-BR were related to reduced MMN amplitudes following an intensity deviant (*r* = .527, *p* = .044).

Regarding MMN latencies, higher SSS-V scores were related to longer MMN latencies following the frequency deviant (*r* = –.504, *p* = .004), and higher BIS scores were related to longer MMN latencies following a location deviant (*r* = .366, *p* = .043) in the full sample. Additionally, higher BIS scores were related to longer MMN latencies following a gap deviant (*r* = –.547, *p* = .035) in the LSTPY group only.

## Discussion

The current study was the first to examine the relationship between schizotypal traits and the MMN using a multi-feature paradigm in a non-clinical sample. This allowed us to explore the MMN in response to multiple auditory deviant types to achieve a more in-depth picture of the effects of schizotypal traits on the early auditory processing system. In line with one previous report (Broyd et al., 2016), but contrary to another (Donaldson et al., 2021), we found no differences between those with high levels of schizotypal traits and those with low levels of schizotypal traits in duration MMN amplitudes. However, we did find significantly reduced MMN amplitudes in response to frequency and location deviants in those with high schizotypal traits. A similar reduction of frequency MMN amplitude has been reported previously in a clinical sample of individuals with SPD (Niznikiewicz et al., 2009), and the current study provides evidence that this deficit may extend to non-clinical samples as well. Accordingly, the current findings support the dimensional conceptualization of psychosis (Peralta & Cuesta, 2007), and provide further evidence that abnormalities in the auditory processing system are present even in the absence of antipsychotic medication.

When examining specific subscales on the SPQ-BR, it was discovered that lower MMN amplitudes in response to frequency deviants were related to higher scores on the “cognitive-perceptual” and “ideas of reference” subscales. Conversely, lower MMN amplitudes in response to a location deviant were related to higher scores on the “social anxiety” subscale. This finding suggests specific aspects of schizotypy may be uniquely related to different components of the early auditory processing system. Specifically, abnormalities in detecting differences in the pitch of incoming auditory stimuli appear to be related to cognitive/perceptual disturbances (i.e., suspiciousness, magical thinking, and unusual perceptions) and ideas of reference (i.e., believing random external events are directly meaningful or related to oneself), while deficits in detecting the perceived location of incoming auditory stimuli appear to be related to feelings of social anxiety. However, previous contrary findings have shown that increased location MMN amplitudes were related to increased general anxiety symptoms in Major Depressive Disorder (Bissonnette et al., 2020) and Panic Disorder (Chang et al., 2015), suggesting there may be something unique about the manifestation of anxiety symptomology in the context of high schizotypal traits and/or social anxiety specifically. Alternatively, the correlations with specific subscales reported here may represent a generalized alteration (i.e., reduction or increase) of sensory perception that co-occurs with these particular sub-traits.

Research thus far has been inconsistent in its reports of MMN abnormalities in individuals with high schizotypal traits (Baldeweg et al., 1999; Broyd et al., 2016; Donaldson et al., 2021; Liu et al., 2007; Niznikiewicz et al., 2009). The current study provides evidence that these varied reports could be, in part, due to the breadth of schizotypal traits in the general population and/or the auditory deviant used to elicit the response. Different samples of individuals with high schizotypal traits may be incredibly diverse in their levels of sub-traits and would therefore contribute to inconsistent findings of abnormalities in schizotypy as a whole if those abnormalities are truly related to certain sub-traits. Furthermore, the auditory change detection system is complex, and the processes required to detect different auditory dimensions can be distinctive (Lee et al., 2017; Phillips & Irvine, 1981; Tiitinen et al., 1993). Therefore, when exploring neuronal markers of schizotypal traits, such as the MMN, examining individual aspects of schizotypy and their effects on the detection of multiple auditory deviants through multi-feature paradigms will allow us to capture more nuanced relationships that may be present.

Finally, we found longer MMN latencies following a location deviant in our high schizotypy group. It should be noted that our high schizotypy group was significantly higher in impulsivity compared to our low schizotypy group, which is not surprising given the previously reported association between impulsivity and schizotypal traits (Del Giudice et al., 2014). However, longer MMN latencies were also significantly related to higher impulsivity and sensation-seeking scores. Therefore, the neurodevelopmental abnormalities that are present in auditory processing may create similarly dysfunctional connections in executive functioning networks that involve greater impulsivity; however, it is impossible to separate the relative contributions of these two colinear factors. In general, those with high levels of impulsivity and sensation seeking may display slower (i.e., less efficient) processing of auditory deviants.

### Limitations and future directions

Limitations to this study include the separation of and low and high schizotypy groups using the continuous variable of SPQ-BR scores which likely reduced our statistical power. Furthermore, the sample used in this study was a fully non-clinical sample, therefore the results of the HSTPY group are generalizable to only those within the general population and not to individuals with clinically significant levels of schizotypal personality traits or schizotypal personality disorder. Additionally, the sample size utilized in this study (*N* = 31) is considered small for investigating individual differences and limits the generalizability of our findings and the robustness of statistical analyses. Specifically, a post-hoc power analysis revealed approximately 50% power was achieved. Accordingly, future research should aim to replicate these findings using larger samples to further validate the observed findings and their implications.

Although recruitment was open to the general public, the majority of our sample were university students which impacts the generalizability of the findings. Finally, although there was not a significant difference in the number of participants in each group that reported a diagnosis of anxiety or depression, and the inclusion of individuals with self-reported diagnoses of anxiety and depression aided in the generalizability of our findings, the presence of anxiety and depression symptoms may have influenced our data. Moving forward, future studies should consider collecting more comprehensive data on the level of current anxiety and depressive symptoms.

### Conclusion

The current study added to the limited body of evidence examining the relationship between auditory change detection (as indexed by the MMN) and schizotypal traits in a non-clinical sample. MMN amplitudes in response to frequency and location deviants were reduced in those with high levels of schizotypal traits. Additionally, differing sub-traits of schizotypy were related to frequency and location amplitudes which may explain previous inconsistencies in the literature. Finally, MMN latencies in response to location deviants were longer in the high schizotypy group, however co-occurring traits of impulsivity and sensation seeking were likely contributing to this difference. Ultimately, the current results provide evidence that abnormalities within the auditory processing system that have been reported in clinical samples of SPD and psychosis also extend to non-clinical populations.
